# Unlocking the Future: Bioprinting Salivary Glands—From Possibility to Reality

**DOI:** 10.3390/jfb15060151

**Published:** 2024-06-01

**Authors:** Dobromira Shopova, Antoniya Yaneva, Anna Mihaylova, Atanaska Dinkova, Desislava Bakova

**Affiliations:** 1 Department of Prosthetic Dentistry, Faculty of Dental Medicine, Medical University-Plovdiv, 4000 Plovdiv, Bulgaria; 2 Department of Medical Informatics, Biostatistics and eLearning, Faculty of Public Health, Medical University-Plovdiv, 4000 Plovdiv, Bulgaria; antoniya.yaneva@mu-plovdiv.bg; 3 Department of Healthcare Management, Faculty of Public Health, Medical University-Plovdiv, 4000 Plovdiv, Bulgaria; anna.mihaylova@mu-plovdiv.bg (A.M.); desislavabakova@gmail.com (D.B.); 4 Department of Oral Surgery, Faculty of Dental Medicine, Medical University-Plovdiv, 4000 Plovdiv, Bulgaria; atanaska.dinkova@mu-plovdiv.bg

**Keywords:** bioprinting, dentistry, xerostomia, salivary glands, secretory organs, bioengineering, stem cells, vascularization, innervation

## Abstract

Salivary gland biofabrication represents a promising avenue in regenerative medicine, aiming to address the challenges of salivary gland dysfunction caused by various factors such as autoimmune diseases and radiotherapy. This review examines the current state of bioprinting technology, biomaterials, and tissue engineering strategies in the context of creating functional, implantable salivary gland constructs. Key considerations include achieving vascularization for proper nutrient supply, maintaining cell viability and functionality during printing, and promoting tissue maturation and integration with surrounding tissues. Despite the existing challenges, recent advancements offer significant potential for the development of personalized therapeutic options to treat salivary gland disorders. Continued research and innovation in this field hold the potential to revolutionize the management of salivary gland conditions, improving patient outcomes and quality of life. This systematic review covers publications from 2018 to April 2024 and was conducted on four databases: Google Scholar, PubMed, EBSCOhost, and Web of Science. The key features necessary for the successful creation, implantation and functioning of bioprinted salivary glands are addressed.

## 1. Introduction

The advent of three-dimensional (3D) and four-dimensional (4D) printing represents a paradigm shift in fabrication methodologies, permeating diverse research domains including engineering, chemistry, biology, computer science, and materials science. Three-dimensional printing facilitates the intricate fabrication of geometries with exceptional accuracy, achieved through the successive deposition of distinct materials layer by layer. The integration of smart materials capable of morphological or color alterations, the generation of electrical currents, the induction of bioactivity, or the execution of pre-defined functions in response to external stimuli heralds the era of dynamic 3D structures, now colloquially termed 4D printing. The application scope of 3D and 4D printing techniques holds considerable promise in scaffold production for tissue engineering, particularly in the custom fabrication of patient-specific scaffolds. Moreover, these methodologies offer avenues for the incorporation of physical and chemical guidance cues into printed scaffolds, thereby enhancing the precision and kinetics of targeted tissue regeneration, Table 1 [1].

Recent advancements have facilitated the three-dimensional (3D) printing of biocompatible materials, cells, and supporting constituents in order to fabricate intricate 3D functional living tissues. This technique, known as 3D bioprinting, is garnering attention in regenerative medicine to address the pressing demand for transplantable tissues and organs. Compared to conventional non-biological printing, 3D bioprinting introduces additional intricacies, including the selection of appropriate materials, cell types, growth factors, and differentiation cues. Technical hurdles associated with the delicate nature of living cells and the assembly of tissues further compound the complexity of bioprinting processes [2,3]. The field of 3D bioprinting is rapidly evolving and is anticipated to revolutionize healthcare in the coming years, with notable potential applications in dentistry [Rodriguez-Salvador]. In the domain of dentistry, 3D bioprinting holds promise as a significant contributor. One area of focus is the bioprinting of salivary glands, employing advanced 3D printing technologies to fabricate functional, living-tissue constructs that emulate the structure and functionality of natural salivary glands. Salivary glands play a crucial role in oral health by producing saliva, which aids in digestion, lubrication, and the protection of the oral cavity. However, dysfunction or damage to these glands, as observed in conditions like xerostomia (dry mouth) in individuals with autoimmune disorders such as Sjögren’s syndrome or those undergoing radiotherapy for head and neck cancers, can profoundly affect an individual’s quality of life [4,5,6,7].

Salivary glands, positioned around the oral and throat regions, derive from the endoderm during embryonic development, comprising acinar and ductal epithelial cells with exocrine capabilities. Classified into major (e.g., submandibular, sublingual, parotid) and minor groups, these glands are primarily situated on the roofs of the mouth and lip, with additional distributions in various throat and laryngeal areas [8,9]. Saliva, an exocrine secretion predominantly composed of water (approximately 99%), harbors a plethora of electrolytes (e.g., sodium, potassium, calcium, chloride, magnesium, bicarbonate, phosphate) and proteins. These proteins include enzymes, immunoglobulins, antimicrobial factors, and mucosal glycoproteins, as well as trace amounts of albumin, polypeptides, and oligopeptides, crucial for oral health. Additionally, saliva contains glucose, urea, ammonia, and other nitrogenous compounds [10,11,12]. Functionally, saliva disperses food throughout the oral cavity, guides taste sensations, and aids in digestion through enzymatic action. Furthermore, it regulates plaque formation, shields tooth enamel, and exhibits protective properties against pathogens, contributing to oral healing processes [13,14,15]. Bioprinting emerges as a promising strategy for regenerating impaired or diseased salivary glands by depositing precise layers of bioink laden with living cells, growth factors, and biomaterials to emulate the native tissue architecture. Researchers are actively exploring diverse bioprinting techniques and materials to fabricate functional salivary gland tissues capable of seamless integration with the host and the restoration of salivary function [16,17]. The intricate challenge in replicating salivary glands lies in recreating their secretory functionality [18].

The purpose of this literature review is to systematize the information about salivary gland bioprinting.

## 2. Materials and Methods

### 2.1. Search Strategy

This review employed the methodological framework for scoping reviews and adhered to the Preferred Reporting Items for Systematic Reviews and Meta-Analyses extension for Scoping Review (PRISMA) guidelines [19]. Invoice Number: INPLASY202450142 Protocol: Unlocking the Future: Bioprinting Salivary Glands—From Possibility to Reality. Date: 31 May 2024 Status: Authorized. The research inquiries guiding this review were as follows: (1) What are the characteristics of bioprinting salivary glands? (2) What are the key fundamental aspects to consider in bioprinting salivary glands?

A comprehensive literature search encompassing publications from 2018 to April 2024 was conducted across four databases: Google Scholar, PubMed, EBSCOhost, and Web of Science. The search strategy incorporated the following terms: (“3D bioprinting” OR “3D-bioprint*” OR “3D print*” OR “Bioprinting” OR “Three-dimensional bioprint*”) AND (“salivary gland” OR “salivary glands”) AND (“Tissue regeneration” OR “Bioengineering”) AND (“Dental” OR “Dentistry”). Additionally, supplementary records were identified through the manual scrutiny of reference lists.

### 2.2. Study Selection

The initial screening of identified reviews involved a meticulous evaluation of titles and abstracts by three independent reviewers. Subsequently, the full texts of potentially suitable studies were retrieved for further assessment against predetermined inclusion and exclusion criteria. Any discrepancies in study selection among reviewers were adjudicated by a fourth reviewer through deliberation.

Inclusion criteria for selected studies encompassed the following: review articles published from 2018 to April 2024, full-text availability, non-payment for access, and publication in the English language.

Conversely, studies were excluded if they constituted case reports, abstracts, paid articles, or conference papers. Furthermore, articles discussing cell biology, bioinks, bioprinting techniques, vascularization, maturation, and innervation in tissues other than the salivary glands were deemed irrelevant to the scope of our review and hence were excluded.

### 2.3. Data Extraction and Analysis

**Figure 1:**
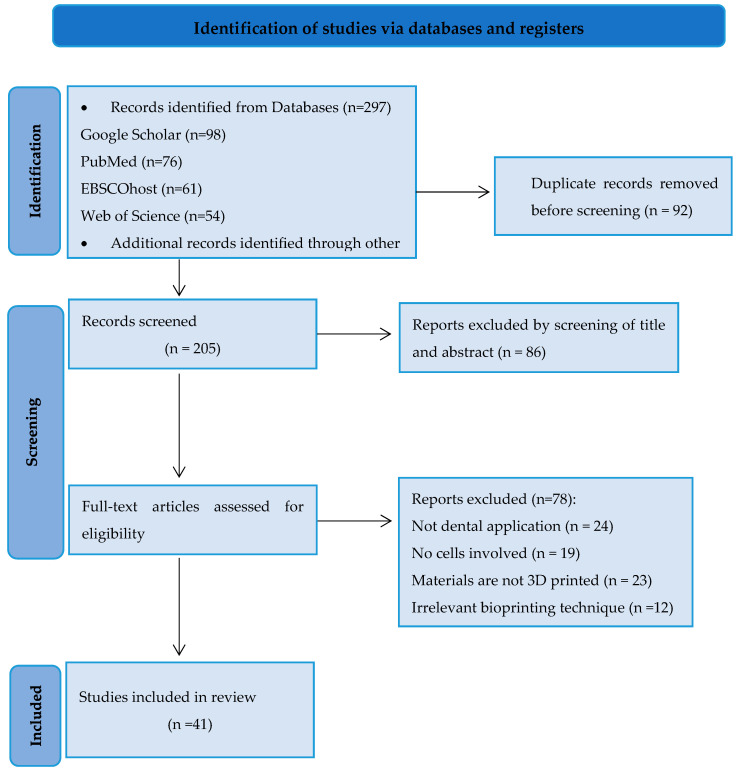
PRISMA flowchart illustrating the selection process of articles for the review.

The first reviewer conducted the extraction and synthesis of information from the included studies, summarizing and organizing the data into a table of evidence. Subsequently, the second reviewer verified the accuracy and alignment of the extracted information with the research questions. The extracted data from the included review articles comprised publication details (including first author and year of publication) and focused on crucial aspects of bioprinting salivary glands, Figure 1.

## 3. Results

Indeed, the field of bioprinting salivary glands is still in its nascent stages, with only 41 articles meeting the established criteria, indicating a significant gap in available information.

From a bioprinting perspective, creating functional secretory organs like salivary glands involves addressing several key aspects to ensure the successful generation of complex, functional tissue constructs. Here are some of the main considerations:

### 3.1. Cell Source and Selection

The identification of suitable cell types is paramount for the successful bioprinting of salivary glands. Various cell populations, including salivary gland epithelial cells, myoepithelial cells, and supporting cells, must be carefully sourced and selected based on their capacity to recapitulate the structure and function of native glandular tissue.

Stem cells are a proven resource in tissue engineering because of their remarkable potential to differentiate into different cell lineages [3,9]. Both adult and embryonic salivary gland-derived cells have demonstrated key in vitro characteristics essential for constructing 3D models of salivary glands [20]. Dental pulp stem cells, classified as adult stem cells, offer distinct advantages as they can be easily obtained from extracted teeth. These cells exhibit multipotency, capable of generating tissues representative of all three germ layers, and possess immunomodulatory properties, rendering them attractive for salivary gland regeneration [21]. Notably, ongoing research focuses on identifying salivary gland stem cells (SGSCs) and multipotent adult stem cells like mesenchymal stem cells (MSCs) for potential regenerative applications. However, recent evidence suggests that the quest for multipotent stem cells within adult salivary glands may be inconclusive [22,23].

Almansoori et al. delineate four primary modalities for 3D cell cultures: spheroids, organoids, 3D microfluidic cell culture systems, and functional decellularized scaffolds. Spheroids derived from oral mucosa have found utility in various oral disease models, with specific gingival spheroids serving as valuable tools for studying gingiva–bacteria interactions [24]. In the realm of salivary gland research, spheroids and organoids have emerged as indispensable platforms for investigating glandular pathophysiology [20]. These 3D culture models provide insights into optimal culture conditions and biomaterials conducive to the organization of dental stem cells (DSCs). Recent investigations utilizing Raman spectroscopy elucidated the role of nutrient diffusion, morphogens, and growth factors in guiding stem cell differentiation and 3D structure formation within spheroids comprising human dental pulp stem cells [25].

Organoids have risen as a promising candidate for considering salivary organ science, showing characteristics associated with local glandular tissues [26]. Salivary organ organoids can be produced through two unmistakable techniques. The primary technique includes actuating pluripotent stem cells (PSCs) to distinguish them into an oral ectoderm inside a 3D culture supplemented with different development variables and cytokines conducive to salivary organ morphogenesis [27]. The other strategy involves the cultivation of salivary organ cells inside 3D platforms, advancing glandular structure arrangement through the activity of development variables [28,29].

Inventive approaches, such as attractive 3D bioprinting frameworks, have encouraged the era of innervated secretory epithelial organoids from dental mash stem cells (DPSCs). These organoids, when transplanted into an ex vivo model, display strong development and innervation, showing high cell reasonability compared to controls. Post-differentiation, the organoids display key salivary organ epithelial compartments, including secretory epithelial, ductal, myoepithelial, and neuronal components. Upon transplantation, the salivary gland-like organoids significantly promote epithelial and neuronal growth within damaged salivary glands, highlighting their regenerative potential [30]. Encouragingly, transplantation success rates are notably high, with 80% of transplanted organoids engrafting at the recipient sites, where organoid ducts seamlessly integrate with host excretory ducts [31].

While allogeneic cell transplantation from healthy donors has been proposed as a potential solution, the definitive identification of salivary gland stem cells capable of glandular regeneration remains elusive. Alternatively, MSCs present an attractive option due to their abundance, well-characterized properties, and established role in signaling crosstalk with the salivary gland epithelium during development and homeostasis. MSCs exhibit trans-differentiation potential and have demonstrated the ability to regenerate salivary gland tissues. However, recent insights suggest that the purported “immuno-privileged” status of allogeneic adult MSCs may not persist post-transplantation. Conversely, autologous MSCs, derived from healthy tissues, circumvent immune challenges and, with advancements in in vitro expansion techniques on tissue-specific matrices, offer a promising therapeutic avenue for restoring salivary gland function [32].

### 3.2. Bioink Development

The development of a bioink formulation that fosters an optimal environment for cell growth, proliferation, and differentiation is imperative in bioprinting salivary glands. The bioink should emulate the composition and functionality of the native extracellular matrix (ECM) while incorporating bioactive molecules, such as growth factors, to enhance cell viability and tissue development.

Bioinks regularly comprise polymers, ceramics, hydrogels, and composites, broadly utilized in tissue design applications [3]. In a perfect world, biomaterials utilized in salivary organ tissue design would have specific properties: supporting cell expansion and relocation inside the lattice, maintaining the phenotypic characteristics of salivary organ cells, advancing the separation of salivary organ stem/progenitor cells, encouraging cell interaction and reorganization (including cell polarization and lumen arrangement), facilitating lattice remodeling (especially in lenient hydrogels), and allowing channel extension to facilitate branching morphogenesis [33].

Various strategies have been devised to culture and study salivary gland cells in vitro, employing diverse hydrogel compositions. Fibrin- and laminin-based hydrogels have demonstrated efficacy in promoting salivary tissue regeneration [34]. The conjugation of laminin-I II trimers with fibrin hydrogels has been shown to enhance the expression of acinar differentiation markers and increase saliva secretion compared to the monomeric form [35]. Fibronectin and placenta basement membrane extract gels have been utilized for the expansion and differentiation of primary human salivary gland epithelial cells in serum-free conditions. Additionally, human salivary spheroids have been successfully cultured using hydrogels composed of egg white and alginate [36].

Responsive materials, though studied for decades, have only recently been tailored for 4D printing applications in tissue engineering. These materials must meet stringent criteria, including biocompatibility, non-cytotoxicity, and preferably biodegradability (resorbability). Moreover, they should possess adequate mechanical strength and be capable of executing dynamic processes within physiological environments. Crucially, the stimuli utilized must be safe and easily controllable when applied in the presence of cells or within the body, necessitating the avoidance of extreme pH values and high temperatures. Consequently, only a select few dynamic polymers fulfill all of these prerequisites [37].

Self-assembled hydrogels speak to a vital category of physical hydrogels, characterized by the unconstrained organization of small particles into the required structures without outside mediation. Different self-assembling molecules, including peptides, recombinant proteins, DNA, small engineered molecules, and copolymers, have been investigated as the building blocks for these hydrogels. Upon introduction into an fluid arrangement, these particles suddenly collect into supramolecular nanostructures. Outstandingly, self-assembled hydrogels show uncommon biocompatibility, focusing on specificity, and biomedical security, rendering them appropriate for the advancement of responsive materials [38].

Within the interest in salivary organ tissue models, careful consideration must be given to the determination of biomaterials and manufacturing strategies to supply a reasonable substrate for cell cultures. A wide range of natural, synthetic, and semi-synthetic materials have been utilized to manufacture platforms, encouraging the examination of the intuitive and empowering implantation of cells in vivo [22].

### 3.3. Bioprinting Technique

**Figure 2:**
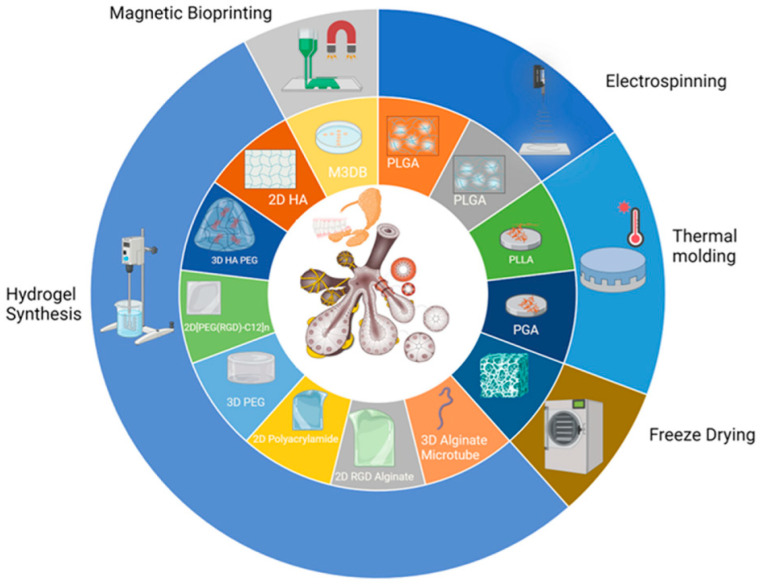
Major approaches to the biofabrication of salivary gland tissues. Reprinted from Ref. [22].

The selection of an appropriate bioprinting technique is pivotal for achieving the desired tissue architecture and cellular organization, Figure 2. Various techniques, such as extrusion-based bioprinting, inkjet-based bioprinting, or laser-assisted bioprinting, may be employed, contingent upon factors such as resolution, speed, and cell viability requirements.

A large number of manufacturing techniques have been investigated within the domain of tissue engineering, encompassing electrospinning, stage division, freeze-drying, self-assembly, improved hydrogels, photolithography, and bioprinting. In any case, a noteworthy challenge within the advancement of built salivary organ tissues for clinical application remains the bioengineering of tissues surpassing 200 µm in thickness that are perfusable and innervated, accurately replicating the inherent characteristics of local tissue [22].

Electrospinning (ES), a customary platform production technique broadly utilized in tissue building, works on the basis of uniaxially prolonging a viscoelastic polymer arrangement or dissolving it under a high voltage [1]. Thermally initiated stage partition (TIPS) empowers the creation of permeable, anisotropic polymer frameworks with controllable structures and negligible losses. In this strategy, a polymer is dissolved at a high temperature, after which it is cooled to actuate stage division and a microporous structure is produced upon solvent removal [39]. Solidifying, combined with water sublimation and the subsequent expulsion of water vapor, has risen as a dependable strategy for standardizing product manufacturing and improving robustness, rendering the item useful for future applications [40].

Gas frothing and water filtering reflect another prevalent platform manufacturing strategy, overcoming the confinements of dissolvable casting by utilizing high weights to liquefy and froth polymers around accurately measured porogens. This strategy includes subjecting a polymer and porogen (regularly sugar or salt particles) to high-pressure carbon dioxide (CO_2_) for an extended period, permitting the consolidation of CO_2_ gas into the polymeric fabric. Upon the controlled discharge of weight, the frothing process guarantees polymer encasement around the porogen, shaping a permeable platform structure. Consequent filtering of the porogen in water yields a sponge-like platform [41].

Inkjet-based bioprinting represents a type of 3D printing technique inspired by traditional desktop inkjet printing methodologies. This approach enables the production of 3D-printed objects in a non-contact manner by depositing ink droplets onto successive layers, tailored specifically for biomanufacturing applications. Drop-on-demand inkjet printing, a prominent variant of inkjet-based bioprinting, can be categorized based on the mechanism through which droplets are ejected. One such mechanism is the thermal approach, wherein liquid is vaporized upon its release from the chamber through the print hole. Alternatively, the acoustic method relies on a mechanical impulse to modulate the shape of a piezoelectric crystal located behind the print head, thereby generating droplets for inkjet printing purposes [42]. Table 2 summarizes the key advantages and disadvantages associated with inkjet-based bioprinting, extrusion-based bioprinting, and light-assisted bioprinting, as described by Yang et al. [43]:

### 3.4. Structural Design

**Figure 3:**
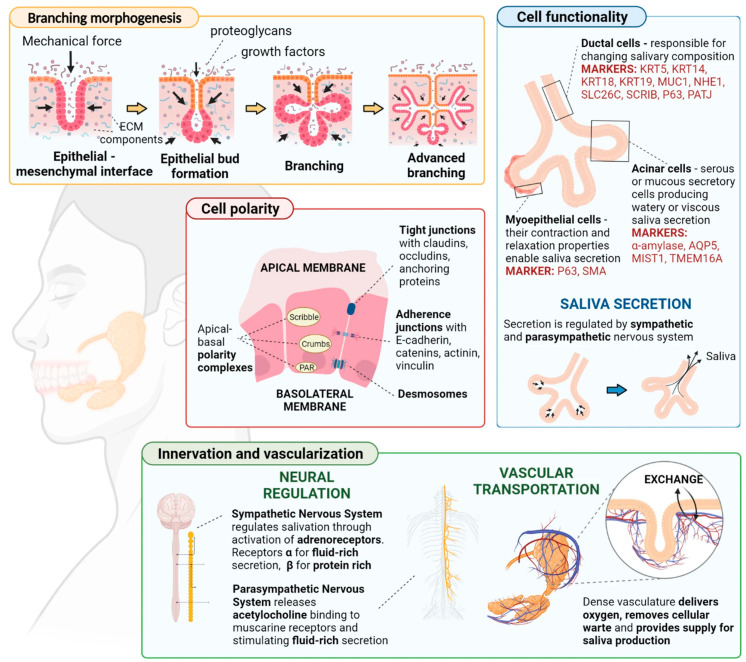
The anatomical and structural features of salivatory glands. Reprinted from Ref. [18].

Designing the tissue construct with the appropriate structural features is essential for mimicking the complexity of native salivary glands. This includes replicating the hierarchical organization of acini, ducts, and blood vessels within the glandular tissue. The anatomical and structural features of salivatory glands are illustrated in Figure 3 [18]:

Additive manufacturing represents a transformative approach to fabrication, especially at the patient-specific level. Computer-aided frameworks utilize modern calculations to plan customized frameworks based on patient-specific images. These frameworks display complex external shapes and spatially designed internal structures, guided by progressed topology optimization methods. The integration of 3D bioprinting and surface alteration strategies improves framework usefulness and osteogenic potential. This is usually accomplished through the consolidation of practical cells, bioactive particles, biomimetic materials, and vectors for transgene expression inside the scaffold’s layered design. By leveraging computational plans, tissue building processes can produce materials with custom-made mechanical, auxiliary, and biochemical properties, clearing the way for personalized regenerative medication approaches [44].

### 3.5. Vascularization

Incorporating a vascular network within bioprinted tissues is paramount to ensuring adequate nutrient and oxygen supply, thereby supporting cell survival and function. Various strategies, such as co-printing endothelial cells or integrating bioactive factors to induce angiogenesis, can be employed to promote vascularization.

The essential work of the vasculature is to supply tissues with oxygen and supplements while encouraging carbon dioxide evacuation. Vasculogenesis, the arrangement of unused blood vessels, includes endothelial cells creating empty capillaries de novo, followed by the enrollment of perivascular wall painting cells (pericytes) and the remodeling of existing blood vessel systems to create a thick vascular plexus. Angiogenesis, the consequent formation of new blood vessels, may be a complex process requiring cooperation between numerous cell types, extracellular lattice components, and development variables. Viable vascularization is vital for appropriate tissue recovery, guaranteeing utilitarian and perfusable vascular systems required for the long-term survival and usefulness of built tissues post-transplantation [45,46,47]. Whereas different vascularization methodologies have been investigated, the significance of microvessel organization inside three-dimensional (3D) frameworks has frequently been ignored. More recent advancements in high-resolution microscopy and image preparation have revealed the profoundly organized nature of microvessels, adjusting themselves with tissue engineering to optimize atomic trade and utilitarian execution [48].

**Figure 4:**
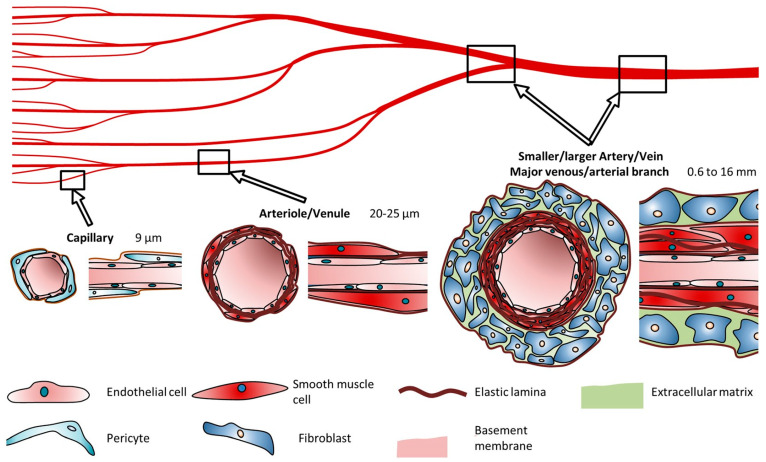
Cell types and composition of vascular channels and their typical diameters according to Schöneberg. Reprinted from Ref. [49].

The number of layers and their thickness within blood vessels depends on their physiological function. Capillaries, with a diameter of approximately 9 µm, consist of a single layer of endothelial cells (ECs) surrounded by pericytes, facilitating rapid and efficient oxygen and nutrient diffusion. Arteries, with a diameter ranging from 0.6 to 16 mm, possess thick, flexible walls capable of withstanding high pressures and accommodating continuous diameter changes. Conversely, veins, with a diameter ranging from 1 to 15 mm, sustain lower blood pressures and exhibit thinner walls lacking the distinct structural organization seen in arteries, Figure 4 [49].

Remodeling is a dynamic and adaptive process inherent in both vasculogenesis and angiogenesis. Endothelial cells play a pivotal role in sensing long-term alterations in the surrounding environment, including changes in growth modulators, vasoactive substances, and inflammatory mediators circulating within the bloodstream. In response to disturbances in the homeostatic balance, endothelial cells adapt by orchestrating structural modifications within the vessel. Initially, the vessel wall perceives changes in hemodynamic conditions such as blood pressure, flow dynamics, or injury. Subsequently, this recognition triggers intracellular communication pathways across various cell layers within the vessel. This communication cascade culminates in the synthesis and release of bioactive molecules. These molecules mediate cellular processes such as proliferation, apoptosis, and migration, as well as the production or degradation of the extracellular matrix (ECM). Consequently, these cellular and acellular components undergo reconstruction or degradation, contributing to the remodeling of the vessel architecture [46,47].

### 3.6. Maturation and Integration

Promoting the maturation of bioprinted tissue and facilitating its integration with the surrounding host tissue are crucial tasks for ensuring long-term functionality. These processes may involve culturing under appropriate conditions to encourage tissue maturation, as well as implementing strategies to enhance host tissue integration upon implantation. Salivary organs start from epithelial branching morphogenesis, which advances through three major stages. Firstly, the improvement of a generally undifferentiated branched structure including acinar and ductal antecedents occurs, followed by the creation of the vasculature and nerves. Then, epithelial branching morphogenesis is actuated by neural crest-derived mesenchymal development components and other atomic signals. Finally, the development preparation occurs, during which the organs ensured to be completely useful and well-differentiated. The innervation and vascularization of the salivary organs progresses in parallel with the arrangement and development of the organs [18,50].

### 3.7. Functionality Assessment

Validating the functionality of bioprinted salivary gland constructs is imperative to ensuring their efficacy in restoring salivary function. This process may entail assessing parameters such as saliva production, protein secretion, and the response to stimuli both in vitro and in preclinical models. The proper functioning of the salivary glands hinges upon adequate innervation. Salivary secretion should increase during feeding and decrease during rest periods. The regulation of saliva secretion involves the interplay of various sensory signals activating afferent fibers of the facial, glossopharyngeal, and trigeminal nerves. Interneurons from the facial and glossopharyngeal nerves transmit signals to the salivary centers. Sympathetic stimulation primarily results in acinar protein-rich secretion, whereas parasympathetic stimulation promotes the production of large volumes of saliva. Crosstalk between the signaling pathways of the main neurotransmitters amplifies saliva flow and protein secretion under normal reflex conditions [18,51,52].

By addressing these main considerations, bioprinting holds the potential to revolutionize the treatment of salivary gland disorders by providing patient-specific functional tissue replacements that can restore normal glandular function and improve quality of life.

Table 3 serves as a synthesis of the pertinent scientific literature, delineating crucial avenues within the realm of salivary gland bioprinting. It serves as a pivotal reference point, furnishing scholars and practitioners alike with a panoramic insight into the contemporary advancements and future trajectories in salivary gland biofabrication.

## 4. Discussion

For patients enduring xerostomia due to hyposalivation resulting from illness or organ damage, a few treatment alternatives exist. In any case, the potential for a consistent arrangement lies in salivary organ substitution through tissue design. More recent achievements have strengthened the vision of making tissue-engineered salivary organs comprising separated salivary epithelial cells capable of shaping useful units that create and provide saliva to the oral cavity. This vision develops our understanding of the cellular components directing branching morphogenesis and salivary epithelial cell polarization in both acinar and ductal structures. By adding development variables and other directional prompts into designed builds, a more complex glandular structure can be created, leading to the close imitation of local salivary organ tissue [53].

To plan a hydrogel appropriate for creating a completely useful multicellular salivary organ, a few key components must be considered. These components include the hydrogel’s composition, the planning strategy, the accessibility of official locales, the firmness, the pore size, push unwinding, and lattice building requirements. Most endeavors in hydrogel-based matrix/scaffold improvement center on making utilitarian salivary organ organoids or spheroids through the self-reorganization of epithelial cells or progenitor cells. This technique leverages central adhesion-mediated signaling with the ECM and high levels of cell–cell interaction, encouraging the arrangement of tight intersections required for salivary organ function [33].

Tissue engineering utilizing stem or progenitor cells holds significant promise for restoring dental and maxillofacial tissues. Dental stem cells (SCs) have emerged as valuable resources due to their capacity for self-regeneration, multidirectional differentiation, and low risk of transplant rejection. Research and clinical applications have demonstrated their utility. However, achieving real and stable tissue regeneration in dentistry requires the integration of various theoretical and technological approaches, including the induction and genetic modification of orofacial SCs [3].

In recent years, stem cell biology has made remarkable strides in the development of -oids (such as gastruloids, spheroids, and organoids) derived from in vitro 3D cultures of SCs. These -oids aim to replicate the physiological properties and tissue architecture of embryonic stages, tissues, and organs [54].

Salivary organ organoid development fundamentally includes two approaches: cultivating salivary gland-derived stem/progenitor cells in a 3D culture framework to imitate regenerative forms and actuating pluripotent stem cells to produce embryonic salivary organs by reproducing their growth process [55].

In spite of the progress made in developing polymeric framework materials for treating dental, oral, and craniofacial damage, accomplishing genuine tissue recovery that combines local morphology, physiological work, and aesthetics remains challenging. Further studies are fundamental to creating polymeric biomaterials with tunable mechanical properties, coordinating debasement rates comparable to physiological remodeling forms, surface functionalization with quality vectors or biologics to upgrade cell properties, and reasonableness for added substance fabrication, including 3D and 4D bioprinting. In the future, polymeric frameworks will play a critical part in personalized care, providing tailored treatment choices to improve clinical results and improve quality of life [34].

While dental stem cells offer numerous applications, they also present certain limitations. One significant challenge lies in the difficulty of identifying, isolating, purifying, and culturing these cells continuously in laboratory settings. Immune rejection poses another obstacle, although the utilization of autologous cells may mitigate this issue. The field of SC research in dentistry faces its own set of challenges and risks, underscoring the need for further investigation [9].

In nature, tissues dynamically respond to environmental changes. While 3D printing facilitates the production of intricate structures for tissue engineering, the resulting constructs remain static and lack the ability to adapt to environmental variations. The advent of 4D printing offers a solution by enabling predicted dynamic transformations in printed structures using responsive materials and/or cells. However, 4D printing is still in the proof-of-concept stage and faces numerous limitations and challenges, including structural design, printing techniques, and ink development. Currently, there is no consistent computational model to accurately predict material transformation over time, necessitating advancements in software and mathematical modeling. Furthermore, cell-based printing techniques are still evolving, and achieving higher resolution in bioprinting remains a challenge due to increased shear forces that can negatively impact cell viability [37,56,57,58].

Vascularization represents a fundamental challenge in tissue building, but recent innovative studies have empowered the advancement of microvascular systems in engineered tissues. Despite the fact that studies are still ongoing, current vascularization strategies show promising results. In vivo models offer the foremost physiologically significant environment for examining tissue improvement and work, whereas progressions in 3D bioprinting innovation improve achievability and accuracy in manufacturing tissues in vitro. Compared to ordinary strategies, 3D bioprinting enables reproducible, adaptable manufacturing with exact 3D control, maintaining high cell reasonability and function [48,59,60].

Successful craniofacial regenerative approaches rely on effective recruitment, regeneration, or integration of both vascularization and innervation. Tissue engineering strategies have been employed to promote vascularization and, more recently, enhance innervation through host tissue recruitment or the prevascularization/innervation of engineered tissues. However, current scaffold designs and approaches for cell or growth factor delivery often fail to coordinate both vascularization and innervation synergistically, hampering successful tissue regeneration. Moreover, vascularization and innervation are typically investigated separately in tissue engineering approaches. Since these tissues work together to improve outcomes in craniofacial tissue regeneration, a revised approach is necessary for the development of engineered materials [61].

Salivary gland-like innervated epithelial organoids and secretomes produced from stem cells offer feasible therapeutic alternatives for SG regeneration. These organoids and secretomes, produced through user-friendly, quick, consistent, and scalable additive manufacturing processes, hold potential for in vitro drug discovery. Bioprinting human SG organoids for drug discovery purposes may reduce the reliance on animal-derived components in tissue constructs and minimize the need for animal experimentation in SG regeneration [62]. Oral organoids are complex three-dimensional structures that are created from stem cells or organ-specific progenitor cells through the process of self-organization and can be used to create models and functionalities comparable to in vivo organs and tissues within the oral and maxillofacial locale. Recently, striking advancements have been made in the development and application of oral organoids of salivary organs. Two fundamental approaches have been utilized to develop salivary organ organoids:

(1) The cultivation of salivary gland-derived stem/progenitor cells in a three-dimensional culture framework to create the structure of the organ through imitating regenerative forms and (2) the actuation of pluripotent stem cells to create embryonic salivary organs by imitating their growth process [55,63].

The salivary organ is composed of a rich epithelial structure that produces saliva and maintains oral homeostasis. Whereas cell lines and creature models have developed our understanding of salivary organ science, they cannot reproduce key characteristics of the human salivary organ tissue, especially the complex engineering and microenvironmental features that contribute to salivary organ function. Organoid communities provide an elective framework to recreate salivary organ tissue in vitro, and salivary organ organoids have been produced from pluripotent stem cells and cultivated stem/progenitor cells [64,65].

## 5. Conclusions

Maintaining cell viability during printing is crucial and can be achieved through the careful selection of bioinks, the optimization of printing parameters, and the incorporation of cell-friendly additives. Additionally, promoting tissue maturation and integration involves creating microenvironments conducive to cell growth and differentiation, as well as facilitating interactions between bioprinted tissues and host tissues. Indeed, bioprinting salivary glands presents several key challenges, including the need for vascularization to ensure adequate nutrient and oxygen supply, maintaining cell viability and functionality throughout the printing process, and promoting tissue maturation and integration with surrounding tissues. However, advancements in bioprinting technology, biomaterials, and tissue engineering techniques offer promising solutions to address these challenges. Efforts to induce vascularization within bioprinted tissues are ongoing, with researchers exploring various strategies such as co-printing endothelial cells or incorporating bioactive factors to stimulate angiogenesis.

Despite these challenges, the progress made in bioprinting technology holds great promise for the development of functional, implantable salivary gland constructs. These constructs have the potential to revolutionize the treatment of various salivary gland disorders, offering patients more effective and personalized therapeutic options in the future. Continued research and innovation in this field are essential to overcome the current limitations and realize the full potential of bioprinting in salivary gland regeneration.

## 6. Limitations

One limitation of the current article is its constrained scope, attributable to inadequate data availability. The analysis relies heavily on theoretical constructs and is hindered by limited empirical evidence. A notable gap in the literature pertains to the dearth of comprehensive scientific studies on salivary gland bioprinting methodologies.

The challenges experienced by researchers result from the following characteristics of SG:

- Salivary organs have a complex microarchitecture, including acini (secretory units), ductal systems, and a rich vascular supply. Precisely imitating this complex structure with bioprinting innovations is highly challenging.
- Obtaining adequate numbers of reasonable cells from salivary organs for bioprinting is troublesome. Essential salivary organ cells are troublesome to culture and the utilization of stem cells requires exact separation techniques to guarantee that they create useful salivary organ cells.
- The choice of bioinks is vital for bioprinting. The improvement and optimization of reasonable bio-inks remains a noteworthy challenge.
- Guaranteeing the satisfactory vascularization of the bioprinted salivary organ tissue is fundamental for its survival and usefulness after implantation. Current bioprinting strategies struggle to create the complex vascular systems required for an adequate blood supply.
- Effectively joining bioprinted salivary organs with existing tissues and providing suitable neural associations for useful discharge and control is another major challenge.
- The host’s immune reaction to the embedded bioprinted tissue can lead to aggravation or dismissal. The immunocompatibility of the bioprinted tissue must be carefully considered.
- Bioprinting for clinical use must go through complex administrative pathways to guarantee security and adequacy. Moral contemplations with respect to the source of cells, particularly in the event that stem cells are utilized, and the long-term impacts of embedded bioprinted tissues require careful consideration.
- Tending to these limitations requires the collaboration between scholars, materials researchers, engineers, and clinicians. Future studies and mechanical investigations are fundamental to overcoming these challenges and realizing the full potential of bioprinting useful salivary organs.

## Figures and Tables

**Table 1:** Comparison of 3D and 4D bioprinting. Reprinted from Ref. [1].

Property	3D Printing	4D Printing
Manufacturing process	2D sections of a 3D structure (with respect to the z-axis) are built layer-by-layer from top to bottom or from bottom to top	Produced in the same way as 3D printed products, but changes shape or function after manufacturing, upon exposure to a specific stimulus
Materials used	Thermoplastic polymers, ceramics, metals, biomaterials, and their composites	Smart materials (polymers, ceramics, metals, biomaterials, and composites) that undergo a change in property or function over time in response to a specific stimulus
Material programmability	Not possible	Material properties and function are programmable with a specific exposure sequence and time of stimulus, and the spatial organization of material in desired final product
Object shape/ function	Stable over time	Object shape or function changes over time when structure is exposed to a specific external stimulus
Application area	Field including but not limited to medical, engineering, dentistry, automotive, jewelry etc.	All 3D print application areas where a dynamic change in configuration is required or beneficial

**Table 2:** Advantages and disadvantages associated with inkjet-based bioprinting, extrusion-based bioprinting, and light-assisted bioprinting. Reprinted from Ref. [43].

Printing Techniques	Resolution	Pros	Cons
Inkjet-based bioprinting	About 100 µm	Low cost; high print speed; High cell survival rate (80–90%)	Low cell viscosity and density; Easily clogged nozzles; Unreliable cell encapsulation
Extrusion-based bioprinting	> 100 µm	Ability to print high cell densities models	Limited resolution; Low print speed; Low probability cell viability
Light-assisted bioprinting	10–50 µm	High resolution, good cell viability (> 95%)	High cost, less efficient

**Table 3:** Significant directions in salivary glands bioprinting.

Key Benefit/Topic	Area of Application/Significance	References
Cell Source and Selection	Adult and embryonic SGs—in vitro SG 3D models	(Pillai et al., 2024) [20]
	SG spheroids and organoids—to study SG pathophysiology	(Pillai et al., 2024) [20]
	The salivary gland-like organoids—stimulated epithelial and neuronal growth	(Adine et al., 2018) [30]
	Autologous MSCs—restoration of SG function	(Marinkovic et al., 2023) [32]
Bioink Development	Ideal biomaterial—cell proliferation and migration, selective differentiation of SG stem/progenitor cells, reorganization, support matrix remodeling, and allow duct expansion	(Pillai et al., 2022) [33]
	Fibrin- and laminin-based hydrogels	(Wu et al., 2021) [34]
	Laminin-I II trimers conjugated with fibrin hydrogels	(Dos Santos et al., 2021) [35]
	Fibronectin and placenta basement membrane	(Zhang et al., 2020) [36]
	Self-assembled hydrogels—biocompatibility, targeting ability, and biomedical safety,	(Chen et al., 2024) [38]
Bioprinting Technique	Freezing	(Andia et al., 2020) [40]
Structural Design	Computational design features—highly tailored mechanical, structural, and biochemical properties	(Latimer et al., 2021) [44]
Vascularization	Co-printing endothelial cells or incorporating bioactive factors—to stimulate angiogenesis	(Nesic et al.) [46] (Tomasina et al.) [47]
Maturation and Integration	Maturation process—fully functional and well-differentiated	(Hajiabbas et al., 2022) [18] (Porcheri et al., 2019) [50]
Functionality Assessment	Neurotransmitter’s signaling—saliva flow and protein secretion under normal reflex conditions	(Hajiabbas et al., 2022) [18] (Khalafalla et al., 2020) [51] (Pedersen et al., 2018) [52]

## Data Availability

Data is contained within the article.

## References

[1] Tamay D.G., Dursun Usal T., Alagoz A.S., Yucel D., Hasirci N., Hasirci V. (2019). 3D and 4D printing of polymers for tissue engineering applications. Front. Bioeng. Biotechnol..

[2] Murphy S.V., Atala A. (2014). 3D bioprinting of tissues and organs. Nat. Biotechnol..

[3] Matichescu A., Ardelean L.C., Rusu L.C., Craciun D., Bratu E.A., Babucea M., Leretter M. (2020). Advanced biomaterials and techniques for oral tissue engineering and regeneration—A review. Materials.

[4] Rodriguez-Salvador M., Ruiz-Cantu L. (2019). Revealing emerging science and technology research for dentistry applications of 3D bioprinting. Int. J. Bioprinting.

[5] von Bültzingslöwen I., Sollecito T.P., Fox P.C., Daniels T., Jonsson R., Lockhart P.B., Wray D., Brennan M.T., Carrozzo M., Gandera B. (2007). Salivary dysfunction associated with systemic diseases: Systematic review and clinical management recommendations. Oral Surg. Oral Med. Oral Pathol. Oral Radiol. Endodontology.

[6] Jensen S.B., Pedersen A.M., Vissink A., Andersen E., Brown C.G., Davies A.N., Dutilh J., Fulton J.S., Jankovic L., Lopes N.N. (2010). A systematic review of salivary gland hypofunction and xerostomia induced by cancer therapies: Prevalence, severity and impact on quality of life. Support. Care Cancer.

[7] Kruszka P., O’BRIAN R.J. (2009). Diagnosis and management of Sjögren syndrome. Am. Fam. Physician.

[8] Yoo C., Vines J.B., Alexander G., Murdock K., Hwang P., Jun H.W. (2014). Adult stem cells and tissue engineering strategies for salivary gland regeneration: A review. Biomater. Res..

[9] Mosaddad S.A., Rasoolzade B., Namanloo R.A., Azarpira N., Dortaj H. (2022). Stem cells and common biomaterials in dentistry: A review study. J. Mater. Sci. Mater. Med..

[10] de Almeida P.D.V., Gregio A.M., Machado M.A., De Lima A.A., Azevedo L.R. (2008). Saliva composition and functions: A comprehensive review. J. Contemp. Dent. Pr..

[11] Humphrey S.P., Williamson R.T. (2001). A review of saliva: Normal composition, flow, and function. J. Prosthet. Dent..

[12] Edgar W.M. (1992). Saliva: Its secretion, composition and functions. Br. Dent. J..

[13] Fatima S., Rehman A., Shah K., Kamran M., Mashal S., Rustam S., Sabir M.W., Muzammal M. (2020). Composition and function of saliva: A review. World J. Pharm. Pharm. Sci.

[14] Dodds M.W., Johnson D.A., Yeh C.K. (2005). Health benefits of saliva: A review. J. Dent..

[15] Kumar B., Kashyap N., Avinash A., Chevvuri R., Sagar M.K., Shrikant K. (2017). The composition, function and role of saliva in maintaining oral health: A review. Proteins.

[16] Adine C., Ferreira J. (2020). Bioprinting strategies to engineer functional salivary gland organoids. Organ Tissue Engineering.

[17] Urkasemsin G., Ferreira J.N. (2019). Unveiling stem cell heterogeneity toward the development of salivary gland regenerative strategies. Stem Cells Heterog. Nov. Concepts.

[18] Hajiabbas M., D’Agostino C., Simińska-Stanny J., Tran S.D., Shavandi A., Delporte C. (2022). Bioengineering in salivary gland regeneration. J. Biomed. Sci..

[19] Sarkis-Onofre R., Catalá-López F., Aromataris E., Lockwood C. (2021). How to properly use the PRISMA Statement. Syst. Rev..

[20] Pillai S., Munguia-Lopez J.G., Tran S.D. (2024). Bioengineered Salivary Gland Microtissues─ A Review of 3D Cellular Models and their Applications. ACS Appl. Bio Mater..

[21] Muallah D., Matschke J., Kappler M., Kroschwald L.M., Lauer G., Eckert A.W. (2023). Dental Pulp Stem Cells for Salivary Gland Regeneration—Where Are We Today?. Int. J. Mol. Sci..

[22] Rose S.C., Larsen M., Xie Y., Sharfstein S.T. (2024). Salivary Gland Bioengineering. Bioengineering.

[23] Rocchi C., Barazzuol L., Coppes R.P. (2021). The evolving definition of salivary gland stem cells. NPJ Regen. Med..

[24] Almansoori A.A., Kim B., Lee J.H., Tran S.D. (2020). Tissue engineering of oral mucosa and salivary gland: Disease modeling and clinical applications. Micromachines.

[25] Kim H., Han Y., Suhito I.R., Choi Y., Kwon M., Son H., Kim H.-R., Kim T.-H. (2021). Raman spectroscopy-based 3D analysis of odontogenic differentiation of human dental pulp stem cell spheroids. Anal. Chem..

[26] y Baena A.R., Casasco A., Monti M. (2022). Hypes and hopes of stem cell therapies in dentistry: A review. Stem Cell Rev. Rep..

[27] TaTanaka J., Ogawa M., Hojo H., Kawashima Y., Mabuchi Y., Hata K., Nakamura S., Yasuhara R., Takamatsu K., Irié T. (2018). Generation of orthotopically functional salivary gland from embryonic stem cells. Nat. Commun..

[28] Sui Y., Zhang S., Li Y., Zhang X., Hu W., Fen Y., Xiong J., Zhang Y., Wei S. (2020). Generation of functional salivary gland tissue from human submandibular gland stem/progenitor cells. Stem Cell Res. Ther..

[29] Farahat M., Kazi G.A., Taketa H., Hara E.S., Oshima M., Kuboki T., Matsumoto T. (2019). Fibronectin-induced ductal formation in salivary gland self-organization model. Dev. Dyn..

[30] Adine C., Ng K.K., Rungarunlert S., Souza G.R., Ferreira J.N. (2018). Engineering innervated secretory epithelial organoids by magnetic three-dimensional bioprinting for stimulating epithelial growth in salivary glands. Biomaterials.

[31] Tanaka J., Senpuku H., Ogawa M., Yasuhara R., Ohnuma S., Takamatsu K., Watanabe T., Mabuchi Y., Nakamura S., Ishida S. (2022). Human induced pluripotent stem cell-derived salivary gland organoids model SARS-CoV-2 infection and replication. Nat. Cell Biol..

[32] Marinkovic M., Tran O.N., Wang H., Abdul-Azees P., Dean D.D., Chen X.-D., Yeh C.-K. (2023). Autologous mesenchymal stem cells offer a new paradigm for salivary gland regeneration. Int. J. Oral. Sci..

[33] Pillai S., Munguia-Lopez J.G., Tran S.D. (2022). Hydrogels for salivary gland tissue engineering. Gels.

[34] Wu D.T., Munguia-Lopez J.G., Cho Y.W., Ma X., Song V., Zhu Z., Tran S.D. (2021). Polymeric scaffolds for dental, oral, and craniofacial regenerative medicine. Molecules.

[35] Dos Santos H., Nam K., Brown C., Dean S., Lewis S., Pfeifer C., Lei P., Petris M., Andreadis S., Baker O. (2021). Trimers conjugated to fibrin hydrogels promote salivary gland function. J. Dent. Res..

[36] Zhang Y., Pham H.M., Munguia-Lopez J.G., Kinsella J.M., Tran S.D. (2020). The optimization of a novel hydrogel—Egg white-alginate for 2.5 D tissue engineering of salivary spheroid-like structure. Molecules.

[37] Saska S., Pilatti L., Blay A., Shibli J.A. (2021). Bioresorbable Polymers: Advanced Materials and 4D Printing for Tissue Engineering. Polymers.

[38] Chen W., Zhang C., Peng S., Lin Y., Ye Z. (2024). Hydrogels in dental medicine. Adv. Ther..

[39] Chocholata P., Kulda V., Babuska V. (2019). Fabrication of Scaffolds for Bone-Tissue Regeneration. Materials.

[40] Andia I., Perez-Valle A., Del Amo C., Maffulli N. (2020). Freeze-Drying of Platelet-Rich Plasma: The Quest for Standardization. Int. J. Mol. Sci..

[41] Rahman S.U., Nagrath M., Ponnusamy S., Arany P.R. (2018). Nanoscale and Macroscale Scaffolds with Controlled-Release Polymeric Systems for Dental Craniomaxillofacial Tissue Engineering. Materials.

[42] Tao O., Kort-Mascort J., Lin Y., Pham H.M., Charbonneau A.M., ElKashty O.A., Kinsella J.M., Tran S.D. (2019). The applications of 3D printing for craniofacial tissue engineering. Micromachines.

[43] Yang B., Liu H., Jiang L., Zeng Y., Han Y., Sha C., Xie X., Li H., Zhou J., Lin W. (2023). 3D bioprinting of collagen-based materials for oral medicine. Collagen Leather..

[44] Latimer J.M., Maekawa S., Yao Y., Wu D.T., Chen M., Giannobile W.V. (2021). Regenerative medicine technologies to treat dental, oral, and craniofacial defects. Front. Bioeng. Biotechnol..

[45] Rademakers T., Horvath J.M., van Blitterswijk C.A., LaPointe V.L. (2019). Oxygen and nutrient delivery in tissue engineering: Approaches to graft vascularization. J. Tissue Eng. Regen. Med..

[46] Nesic D., Durual S., Marger L., Mekki M., Sailer I., Scherrer S.S. (2020). Could 3D printing be the future for oral soft tissue regeneration?. Bioprinting.

[47] Tomasina C., Bodet T., Mota C., Moroni L., Camarero-Espinosa S. (2019). Bioprinting Vasculature: Materials, Cells and Emergent Techniques. Materials.

[48] Sharma D., Ross D., Wang G., Jia W., Kirkpatrick S.J., Zhao F. (2019). Upgrading prevascularization in tissue engineering: A review of strategies for promoting highly organized microvascular network formation. Acta Biomater..

[49] Schoeneberg J., De Lorenzi F., Theek B., Blaeser A., Rommel D., Kuehne A.J.C., Kiessling F., Fischer H. (2018). Engineering biofunctional in vitro vessel models using a multilayer bioprinting technique. Sci. Rep..

[50] Porcheri C., Mitsiadis T.A. (2019). Physiology, Pathology and Regeneration of Salivary Glands. Cells.

[51] Khalafalla M.G., Woods L.T., Jasmer K.J., Forti K.M., Camden J.M., Jensen J.L., Limesand K.H., Galtung H.K., Weisman G.A. (2020). P2 receptors as therapeutic targets in the salivary gland: From physiology to dysfunction. Front. Pharmacol..

[52] Pedersen A.M.L., Sørensen C.E., Proctor G.B., Carpenter G.H., Ekström J. (2018). Salivary secretion in health and disease. J. Oral Rehabil..

[53] Barrows C.M., Wu D., Farach-Carson M.C., Young S. (2020). Building a functional salivary gland for cell-based therapy: More than secretory epithelial acini. Tissue Eng. Part A.

[54] Eisenstein M. (2018). Organoids: The body builders. Nat. Methods.

[55] Gao X., Wu Y., Liao L., Tian W. (2021). Oral organoids: Progress and challenges. J. Dent. Res..

[56] Miao S., Zhu W., Castro N.J., Nowicki M., Zhou X., Cui H., Fisher J.P., Zhang L.G. (2016). 4D printing smart biomedical scaffolds with novel soybean oil epoxidized acrylate. Sci. Rep..

[57] Yang Q., Gao B., Xu F. (2020). Recent advances in 4D bioprinting. Biotechnol. J..

[58] Ionov L. (2018). 4D biofabrication: Materials, methods, and applications. Adv. Healthc. Mater..

[59] Xu X., Liao L., Tian W. (2022). Strategies of prevascularization in tissue engineering and regeneration of craniofacial tissues. Tissue Eng. Part B Rev..

[60] Sasmal P., Datta P., Wu Y., Ozbolat I.T. (2018). 3D bioprinting for modelling vasculature. Microphysiol. Syst..

[61] Li Y., Fraser D., Mereness J., Van Hove A., Basu S., Newman M., Benoit D.S. (2021). Tissue engineered neurovascularization strategies for craniofacial tissue regeneration. ACS Appl. Bio Mater..

[62] Chansaenroj A., Yodmuang S., Ferreira J.N. (2021). Trends in salivary gland tissue engineering: From stem cells to secretome and organoid bioprinting. Tissue Eng. Part B Rev..

[63] Li Z., Yue M., Liu Y., Zhang P., Qing J., Liu H., Zhou Y. (2022). Advances of engineered hydrogel organoids within the stem cell field: A systematic review. Gels.

[64] Zhao C., Meng C., Cui N., Sha J., Sun L., Zhu D. (2021). Organoid models for salivary gland biology and regenerative medicine. Stem Cells Int..

[65] Calà G., Sina B., De Coppi P., Giobbe G.G., Gerli M.F.M. (2023). Primary human organoids models: Current progress and key milestones. Front. Bioeng. Biotechnol..

